# A Database of Stress-Strain Properties Auto-generated from the Scientific Literature using ChemDataExtractor

**DOI:** 10.1038/s41597-024-03979-6

**Published:** 2024-11-23

**Authors:** Pankaj Kumar, Saurabh Kabra, Jacqueline M. Cole

**Affiliations:** 1 Cavendish Laboratory, Department of Physics, University of Cambridge, J. J. Thomson Avenue, Cambridge, CB3 0HE UK; 2 ISIS Neutron and Muon Source, STFC Rutherford Appleton Laboratory, Harwell Science and Innovation Campus, Didcot, OX11 0QX UK; 3 Research Complex at Harwell, Rutherford Appleton Laboratory, Harwell Science and Innovation Campus, Didcot, Oxfordshire OX11 0FA UK; 4 Present Address: Neutron Sciences Directorate, One Bethel Valley Rd, Oak Ridge, TN 37831 USA

**Keywords:** Mechanical engineering, Materials for devices, Mechanical properties, Metals and alloys

## Abstract

There has been an ongoing need for information-rich databases in the mechanical-engineering domain to aid in data-driven materials science. To address the lack of suitable property databases, this study employs the latest version of the chemistry-aware natural-language-processing (NLP) toolkit, ChemDataExtractor, to automatically curate a comprehensive materials database of key stress-strain properties. The database contains information about materials and their cognate properties: ultimate tensile strength, yield strength, fracture strength, Young’s modulus, and ductility values. 720,308 data records were extracted from the scientific literature and organized into machine-readable databases formats. The extracted data have an overall precision, recall and F-score of 82.03%, 92.13% and 86.79%, respectively. The resulting database has been made publicly available, aiming to facilitate data-driven research and accelerate advancements within the mechanical-engineering domain.

## Background & Summary

The understanding of material behaviour and design forms the foundation of numerous technological advancements and applications across diverse industries, cementing the importance of materials science. Whether it be aerospace, civil or automotive engineering, the selection and design of materials are crucial determinants of product performance, safety and longevity^1–3^. In particular, stress-strain properties, which describe the response of a material subject to mechanical loads, give insight on the ability of a material to withstand deformation, fracture and fatigue^4,5^. As such, successful projects in engineering-related domains require careful consideration of these stress-strain properties.

However, traditional research workflows used to study stress-strain properties often rely on a time-consuming and iterative process of experimentation and modelling. This approach can be inefficient, destructive and costly, limiting advanced study of material properties and behaviour, thus preventing the rapid development and optimization of new materials^6^. In recent years, data-driven research has emerged as an alternative workflow which leverages extensive datasets and machine learning algorithms to enable a deeper study of materials and accelerate their discovery timeline^7^.

The successful implementation of data-driven techniques in materials science hinges on the availability of high-quality, comprehensive, and readily-available material data^8^. While substantial progress has been made in supplying datasets suitable for this new type of research in other domains of materials science^9,10^, there remains a significant gap in the availability of open-source stress-strain property data for materials engineering. Some have turned to the development of natural-language-processing (NLP) tools and information-extraction pipelines to aid in database creation by targetting the published scientific literature^11^. For example, the creation of the materials-aware NLP toolkit, ChemDataExtractor^12–15^, has enabled the automatic generation of material databases for various application areas, e.g. thermoelectrics^16^, optics^17^, magnetism^18^, semiconductors^19^, batteries^20^, photovoltaics^21^ and optoelectronics^22^. ChemDataExtractor has also been adapted for information extraction in the engineering sector, the first attempt being the construction of a materials database about yield strength and grain size^23^.

This study targets the curation of a comprehensive materials database of mechanical properties that typically feature on a stress-strain curve: yield strength, ultimate tensile strength, Young’s modulus, fracture strength and ductility. Through an improved information-extraction pipeline, a database of 720,308 material properties with an overall precision of 82.03% has been presented. This database is ten times larger than our previous work^23^ and contains data records about four additional stress-strain property characteristics. To the best of our knowledge, this is the largest public repository of experimental data on stress-strain properties that have been curated from the scientific literature for a wide range of materials. The *Methods* section will outline the information-extraction pipeline that was used in this work. Descriptions of the resulting database and its evaluation is provided in the *Data Records* and *Technical Validation* sections, respectively.

## Methods

A database was generated for the following mechanical properties: yield strength, ultimate tensile strength, Young’s modulus, fracture strength and ductility, all of which are defined features of a stress-strain curve. All data were mined from the scientific literature using the ChemDataExtractor codebase^15^, which was subjected to bespoke adaptations for handling domain-specific materials-engineering information for this study. This bespoke version of the codebase, ChemDataExtractorStressEng, is herewith made available on https://github.com/gh-PankajKumar/chemdataextractorv2.3-stresseng. The overarching database-generation process is similar to that of the previously developed materials database that contains only yield-strength properties^23^, in that it consists of three steps: corpus acquisition, information extraction and post-processing. However, there are some significant differences. In particular, the article retrieval process in this work includes data-extraction from Springer Nature publications as well as papers from Elsevier. Moreover, broader search parameters were used to target the retrieval of relevant documents from these sources of text to form a larger corpus. Additionally, the property-model module that is now available in ChemDataExtractor, version 2, was used to accommodate the new target properties. These significant differences in data extraction will be highlighted in the following sub-sections.

### Corpus acquisition

The corpus for this study was built using downloadable content published on Elsevier and Springer Nature as these are two of the largest academic publishing bodies with text and data-mining policies that allow for the mass scraping of scholarly articles, including research articles, reports and books, through an API. This enables efficient access to their repositories, and by filtering with specific queries, curated literature can be retrieved in a consistent format that is ready to be used for information extraction.

Communication with each Application Programming Interface (API) was handled programmatically. Webscraping tools were built using the Python libraries *Requests* and *BeautifulSoup* that search and download content in volume. For both publishers, queries were constructed to search for content which contained some mention of the mechanical properties in the text body. An example search query would be: *“yield strength“OR“ultimate tensile strength“OR“young’s modulus”*. Here, search operators were employed to further narrow the focus; the use of the quotation marks ensured that the contained string appeared exactly as written and the *OR* operator allowed for multiple queries to be grouped together. A limit of 6,000 and 100 articles per request for a given set of parameters is imposed by Elsevier and Springer, respectively. Therefore, each request was constructed with additional parameters, such as page number or publication year, which were iterated over to gather a larger number of articles than a single API request can return for a given search query.

First, the relevant Document Object Identifiers (DOIs) returned from a search request were extracted from the API response. For Elsevier, the response body can be read as a JavaScript Object Notation (JSON) formatted document such that the result of a search is a list of dictionaries containing article data. As such, the DOI of articles were extracted from these dictionaries and stored for downloading. In contrast, Springer returns an eXtensible Markup Language (XML) response with article data stored within XML tags. Therefore, DOIs of articles sourced from Springer were extracted using *BeautifulSoup* to find tags that contain the necessary information; for instance, nested under an article-id tag, “pub-id-type”: “doi” would contain the DOI. A total of 480,738 articles were found that matched the search criteria with 347,820 from Elsevier’s API and 132,918 from Springer Nature’s API.

The article text associated to each DOI was then requested from the corresponding API and the response was saved in XML format. To avoid issues further down the information-extraction pipeline, articles were filtered and sorted. Firstly, articles that were not complete were filtered out and only full-text content was preserved. For Elsevier, these were identified with a subtype tag “fla”. Full text articles from Springer Nature were identified if multiple section tags that contain text were present as these denote the sections in an article. During this process, metadata were extracted from the articles that will be used to supplement the final database to give more context to each record.

In the filtering process, 39,662 and 58,849 articles published by Elsevier and Springer Nature were discarded, respectively. Over half of those discarded Elseiver articles included book chapters or short communications since our focus for extraction was full research articles. At first sight, it may seem surprising that such a large number of Springer Nature articles were filtered out, especially since their majority had been classified as research articles during the search process. Yet, over half of these articles were published before 2005 and 12,406 of them had been published before 1990; this implies that they may only be available as Portable-Document-Format (PDF) files and therefore, their full text content was not included in the XML response. The total number of articles that formed the corpus is listed in Table 1.

**Table 1:** Summary of the corpus size after article retrieval, partitioning the research articles into those available via subscription (Sub) and those via open access.

Publisher	Sub Access	Open Access	Total
Elsevier	278,124	30,034	**308,158**
Springer	61,789	12,280	**74,069**
Total	339,913	42,314	**382,227**

**Fig. 1:**
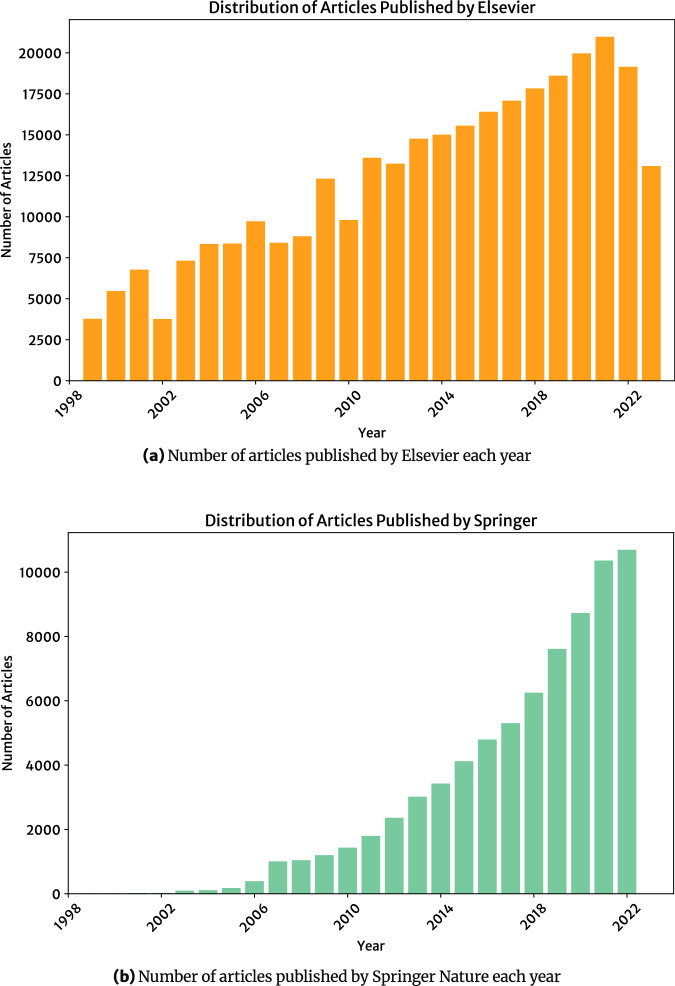
Yearly distribution of articles pertaining to stress-strain information that were retrieved using the Webscraping tools that form the corpus of this work which showcases the exponential growth of research output in the overarching materials engineering field.

The distribution of articles and publication year for each publisher is shown in Fig. 1. An interesting feature of this yearly distribution is that they show the exponential growth of research output. The yearly distribution of published Springer Nature articles reveals that publications in materials engineering have been growing at a particularly high rate which highlights the importance of auto-generating a property database in this field for data-driven materials research. The filtered corpus was then prepared for information extraction, in the same fashion as that described in previous work^23^.

### Information extraction

**Fig. 2:**
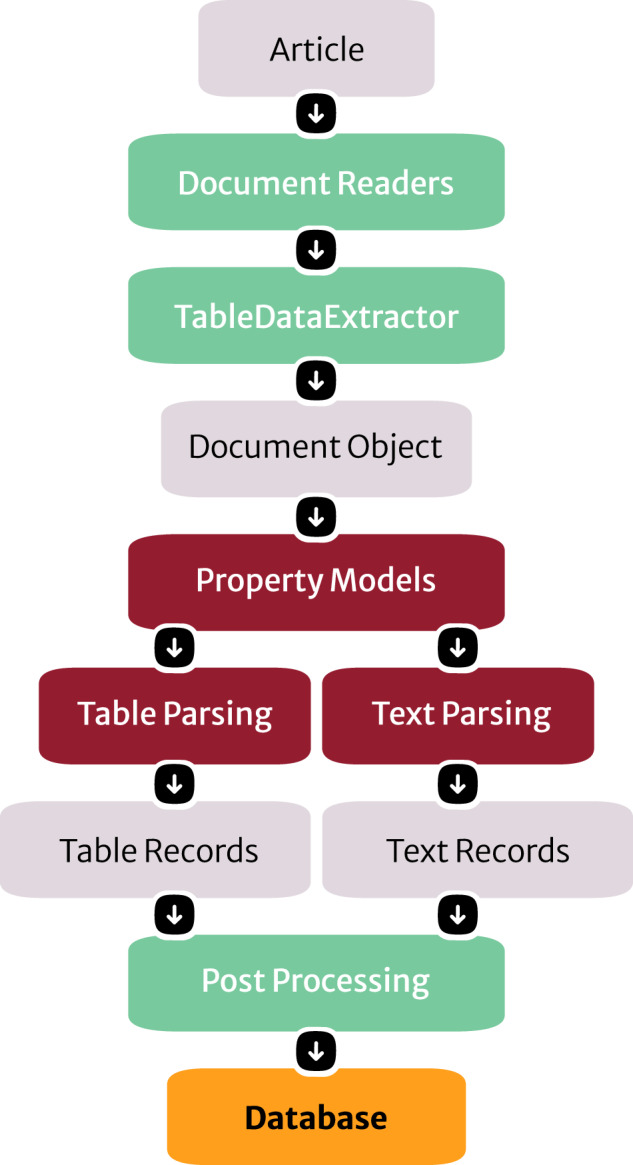
The information-extraction pipeline used in this study. ChemDataExtractor readers process input articles, converting them into *Document* objects which are representations of an article that are suitable for the information extraction pipeline. Each object is assigned a set of property models that assign extraction parsers and define output data record structure. Post-processing organizes the extracted text and table records to ensure data validity before database storage.

Figure 2 presents the information-extraction pipeline that was used in this work. Firstly, an XML article from the corpus was processed using the *reader* package in ChemDataExtractor. This handles the conversion from XML to plain text and then into a *Document* object, an optimized representation of the input full-text article and all its components^13^. The *Elsevier* and *Springer JATS* readers were used to handle the specific XML formatting conventions, determine the article structure, gather the full text, and clean the file from unnecessary tags that might hinder the information-extraction process. To handle tabulated data, an additional step was taken where by the TableDataExtractor implementation within ChemDataExtractor^13^ was used to convert tables into a standardized format. This is necessary because tables often consist of complex structures with nested rows and columns that could cause difficulties for accurate parsing. The *Document* objects were then passed through the pipeline for processing where they were assigned to property extraction models.

These models define the methods of information extraction and the desired structure of an output data record. Two models were created for each mechanical property: one for table data extraction and one from textual prose. Details of the property extraction models used in this study will be given in subsequent sections. With these assigned property models, the *Document* was processed, extracting relevant information from the text and table content to generate data records. Those data records that had a complete knowledge representation, whereby each field in the property model had a valid value, were stored separately as JSON files.

In practice, the information-extraction pipeline up to this point was parallelized using a Message Parsing Interface (MPI), such that multiple articles could be processed simultaneously. Data extraction was performed using the Cooley supercomputing resources of the Argonne Leadership Computing Facility, a U.S. Department of Energy (DOE) Office of Science user facility at Argonne National Laboratory, Illinois, USA. The Cooley supercomputer consists of 126 computing nodes, each equipped with two 6-core, 2.4 GHz Intel E5-2620 processors and one NVIDIA Tesla K80 GPU.

The final stage of information extraction involved data post-processing, in which the extracted data records from text and table were combined and converted into formats suitable for data-driven research. All JSON files were imported into a single *Pandas* dataframe^24,25^ since this allows for exporting to multiple formats easily. The dataframe contains the following columns: *Record Type, Specifier, Compound, Raw Value, Raw Units, Normalized Value, Normalized Units, Article*. The normalization of property values and units follows the method used in our previous work^13^; stress units are converted to the standard form, Pascals, and the corresponding conversion is applied to the property value.

In other work, additional filtering to the data records may be carried out; for example, there are instances of compound names being filtered using the Material Parser^26^ or by employing a blocklist^23^. While these techniques can be useful, the broader reach of the corpus in this study, and consequently, its greater diversity in extracted compound names, means that there are potentially more correct data records that would be flagged as false positives if one were to use the Material Parser. Therefore, the metadata retrieved during the corpus acquisition in this work was added to each data record. Besides, the metadata are more useful than a simple blocklist as they provide the end user with the ability to make more informed decisions on how they interpret the data. Furthermore, the included metadata allows the resulting database to be filtered using custom methods, to derive more specialized subsets of data. For example, data records could be filtered according to the journal in which they are reported, affording database subsets that are specific to a given subdomain of interest. This approach differs from our previous work on this topic^23^, where a specialized “engineering-ready” database was derived, using a filter on yield-strength value which is very lenient and does not accurately represent a particular domain within material science.

The processed data were exported in *CSV* and *JSON* files as these formats are platform-independent and widely supported by different programming languages and tools. This universality enables the databases to be compatible with a variety of data-analysis tools, libraries and frameworks, which in turn will facilitate data-driven materials stress-strain property research.

While the general implementation of the information-extraction pipeline is similar to that which we previously employed^23^, this work introduces key modifications to the Chemical Named-Entity Recognition (CNER), Property Models, and Record Parsing resulting in an improved extraction pipeline. These changes are detailed in the following section.

### Chemical Named-Entity Recognition (CNER)

**Fig. 3:**
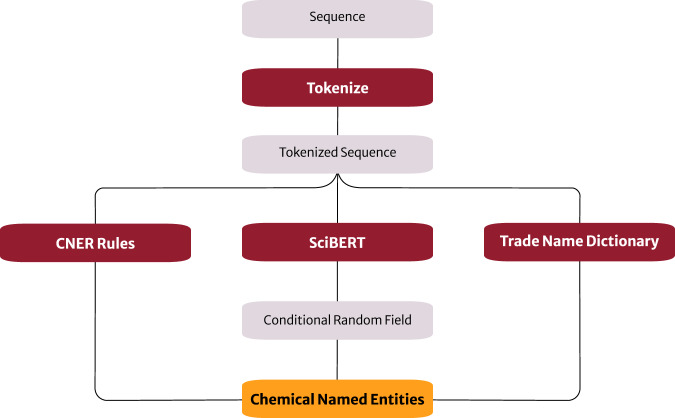
The pipeline of the Chemical Named Entity Recognition (CNER) method used for information extraction. Built-in methods of ChemDataExtractor tokenize the input word sequence. Chemical entities are then identified using one of the three methods: custom CNER rules, SciBERT, or matching tokens to a predefined trade name dictionary.

The original ChemDataExtractor (version 1)^12^ used a combination of rule, dictionary and conditional-random-field-based methods for CNER tasks. On a small, focused corpus, these methods can be tuned with additional rules and dictionary entries to achieve good performance in CNER, as demonstrated by Kumar *et al*.^23^ through the generation of a materials database for yield-strength properties^23^. In that work, the specifically designed rules and dictionaries of trade-names enabled the handling of chemical entities within the engineering subdomain. However, it is difficult to craft CNER rules to account for all the additional material name possibilities that arise when using a larger and broader corpus, as is used in this study. The CNER system employed in this work therefore comprised a more sophisticated process, the workflow for which is illustrated in Fig. 3. This consists of the latest CNER-based algorithms that are publicly available within the ChemDataExtractor codebase^14^ (the inner paths in Fig. 3), together with rule-based and dictionary-based methods (the two outer paths in Fig. 3) that have been specifically designed for this work.

The CNER-based developments by Isazawa and Cole^14^ employ a language model to assist CNER, which is built on the Bidirectional Encoder Representations from Transformers (BERT) architecture (*c.f*. Vaswani *et al*.^27^ and Devlin *et al*.^28^). Specifically, they fine-tuned the SciBERT language model^29^ for CNER using manually annotated training data including both organic and inorganic named chemical entities. SciBERT is an implementation of the BERT model whereby it was pre-trained on the Semantic Scholar Corpus^30^, i.e., a large collection of papers that spans all fields of science. Its embedded scientific knowledge was deemed to make it more useful than the foundational BERT model for downstream tasks in scientific fields, such as CNER. The tokenizer that feeds into SciBERT in our CNER pipeline (shown in Fig. 3) was created as an integral part of the developments by Isazawa and Cole^14^. The associated tokenized sequence that feeds into the fine-tuned SciBERT model detects chemical entity tags, which are validated using a Conditional Random Field. The fine-tuning process uses a training corpus of abstracts with annotated chemical entities. The corpus is a combination of data from the CHEMDNER^31^ and Matscholar^32,33^ datasets, i.e., it spans both organic and inorganic materials. This chemical breadth and materials-domain diversity aligns with that of the corpus gathered in this study. The learnt adaptability of this language-model-based CNER method therefore stands to be useful for information extraction herein.

The rule-based and dictionary-based methods, that have been specifically designed to detect materials common in the materials-engineering subdomain, supplement the SciBERT-based component of the CNER workflow by trying to detect chemical mentions that it may have missed. On the one hand, the tokenized sequence is scanned using *CNER Rules* that comprise regular expressions to detect alloy compositions which may span over more than one token. On the other hand, a dictionary of commonly used trade names immediately detects matching tokens and these are labelled as chemical entities; *cf. Trade Name Dictionary* in Fig. 3. While these rule-based and dictionary-based methods follow closely to those described previously^23^, they have been modified to be less computationally intensive for this work. For optimal performance, the *CNER Rules* were segmented into the smallest possible function while the *Trade Name Dictionary* was divided into smaller components so that these components could be processed in small chunks. This reduced the computational processing time of a research article when subjected to the rule-based and dictionary-based CNER methods by almost a third, compared with the foundational CNER functionality of ChemDataExtractor version 2.0^13^ employed in our previous work^23^.

### Property models

The operational pipeline of ChemDataExtractor is set up such that the extraction methods and desired structure of an output record are defined in a *Property Model*. To reiterate, a property model can be understood as a blueprint for how ChemDataExtractor should interpret and extract relevant information in a scientific document. For example, a *YieldStrengthModel* would define how to identify, extract, and store yield strength properties. These models can be built in modular fashion such that representations of properties in ChemDataExtractor are defined using a set of simple base models such as those that handle compounds or specific units. Further details on the implementation of property models and their concept is given by Mavračić *et al*.^13^. As mentioned, two property models, one for text extraction and one for table extraction, were created for yield strength, ultimate tensile strength, Young’s modulus, ductility and fracture strength. The property models were built using extensions to the ChemDataExtractor toolkit from our previous work^23^, affording the foundational *StressModel* that enables the parsing of stress-strain characteristics of materials. An example property model is given in *code snippet* 1.

The different components of each property model and their function are given in Table 2. Each property model inherits from some form of a base model that defines the handling of values and units or compounds and their name. These are constructed using components to define how to extract and represent relevant information in a modular fashion. Four of the five properties were reported with units of stress, and they thus inherited the *StressModel*^23^. The other property, ductility, was mostly reported as a percentage for which the *RatioModel*, included in ChemDataExtractor version 2.0^13^, was used. Each property model contains a *specifier* that was constructed using rules defined by regular expressions. This specifier was used to identify any mention of the property in the article text during the information-extraction process. At the most basic level, the name of the property itself was used for the specifier. However, a property is often portrayed as an acronym or a mathematical symbol, such as *σ*_*ys*_ for yield strength, instead of its property name. Therefore, the specifier also contains regular expressions that served as parsing rules for the most common symbols, determined by an initial review of engineering text books and the scientific literature, to identify each property. Moreover, each property model contains a *CompoundModel* which represents the relevant compound and contains the CNER methods that have been discussed earlier.

**Table 2:** The necessary components when defining property models in this work.

Component	Function
specifier	Regular expression rules used to identify a property mention.
compound	A base model used to identify relevant compound names and labels. This makes use of the CNER methods that have been described.
raw_value	The value of a property as it appears in text. This is required for a complete record.
raw_units	The units of a property as it appears in text. This is required for a complete record.
parsers	The parsing methods that were used for relation association and subsequent data extraction. For text models, the extended *AutoParser* in ChemDataExtractor version 2.0 was used. For table models, the *AutoTableParser* was used in ChemDataExtractor version 2.0.

**Code Snippet 1**. Example Property Model for ultimate tensile strength built using the base StressModel.
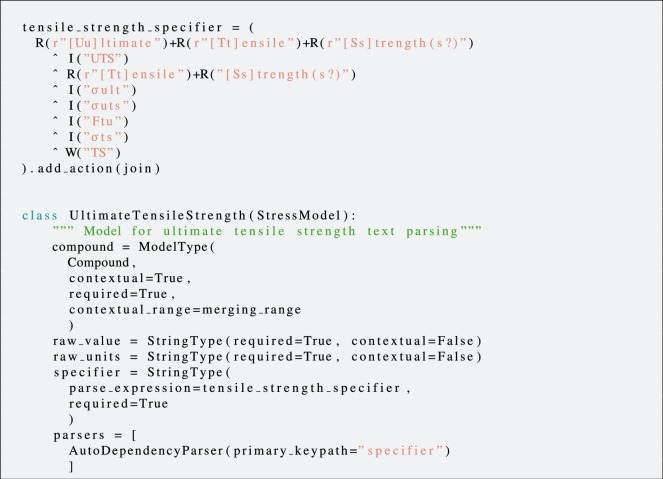


### Record parsing

Given the large number of property models in this study, the *AutoParsers* in ChemDataExtractor version 2.0^13^ were used to extract data owing to their ease of set up. These *AutoParsers* use predefined grammar rules to extract structured property information from unstructured text. The high level of semantic similarity, when discussing different mechanical properties, gives good reason for the application of predefined grammar rules and *AutoParsers*.

For tabulated data that have been standardized, the *AutoTableParsers* in ChemDataExtractor version 2.0^13^ worked well “out-of-the-box”, achieving high performance without explicitly defining parsing rules owing to their highly structured nature. This automatic table parser scans each row and extracts any relevant information determined by the property model. The extracted information is consolidated into a complete record, merging around the table information, such as the table caption, which may contain relevant information such as the chemical entity.

Similarly, for textual-prose data, the automatic text parsers of ChemDataExtractor v2.0^13^ did not tend to require one to define new parsing rules for each model. Yet, it should be borne in mind that the unstructured nature of text may lead to worse performance when using the auto-parsers in ChemDataExtractor v2.0, in contrast to the situation for automatic table parsing. To illustrate this, we consider the case where the same units are used for multiple properties in a single sentence of text; the automatic text parser may struggle to correctly identify the relationships between value, unit, property and material. For instance, the sentence, *“Material X has a yield strength and ultimate tensile strength of* 250* MPa and* 300* MPa respectively”* may lead to the yield strength or ultimate tensile strength being incorrectly extracted with an incorrect value or a duplication. This challenge has been addressed by Isazawa and Cole^15^. They introduced the DepIE algorithm to correctly associate spans of text for use in extracting photocatalysis information. The algorithm was initially used for more intricate property-model structures, with nested models representing complex relations. However, it can serve a more general purpose.

Thereby, this study used the DepIE algorithm to help to correctly associate stress-related quantities with their corresponding specifier within a single relevant property model. The algorithm takes an element defined in the property model as an input, which is necessary for a record to be identified and this is termed “key field”. Here, the key field is set as the specifier that is used to identify the existence of a record for each text property model. The CNER methods and rule-based parsing of ChemDataExtractor are then used to determine relevant spans of text of the same type. For instance, in the example sentence given above: there is one key field (*“yield strength”*) and two relevant value fields that would be identified by this method (*250* and *300*). The distance from the key field to the identified relevant text spans on a syntactic dependency graph^34^ is then calculated. This process is repeated for all fields defined in the property model. By minimizing the dependency graph distance, complete data records are extracted from the source text, whose contents have a higher likelihood of being correctly associated. A dependency graph is used since the actual distance in a sequence may not accurately show closely related text spans.

Additionally, each association of text span is assigned a confidence score. This is calculated using: *D*_*min*_, which is the distance between the key field and the most closely associated text span; and *D*_*second*_, which is the distance between the key field and the second most closely associated text span, for a given field. Mathematically, this is given as^15^:

1 $${\rm{Confidence}}=max\left\{\frac{1}{\frac{{D}_{min}}{{D}_{second}}+1},\frac{1}{{n}_{key}{n}_{span}}\right\}$$

where *n*_*key*_ is the number of key fields and *n*_*span*_ is the number of text spans for a particular field. The two terms can be conceptualized as probabilities: the first term is an approximation of the probability of a correct association with DepIE and the second term is the probability of a correct association chosen at random. Intuitively, if *D*_*min*_ and *D*_*second*_ are the same, then probability of the algorithm choosing the correct association would be the same as a random assignment. While, if *D*_*min*_ is much smaller than *D*_*second*_, then the probability of the algorithm finding the correct association is much higher than that of random chance. This confidence score is used to assist in merging records across different sentences and supplements the merging methods used in ChemDataExtractor versions 1 and 2.0^12,13^. More details of this DepIE implementation are given by Isazawa and Cole^15^.

## Data Records

This automated information-extraction process resulted in a materials database of stress-strain properties that contains 720,308 data records, whose nature is characterized by the database keys detailed in Table 3. The type of data record is labelled using the property model that was employed for the information-extraction process; as such, it will identify whether the data record originates from textual prose or tabular content of a research article. The specifier of the property that was found when parsing the information captured by the data record is provided exactly as it appears in the source. Each data record contains a *Compound* list whose details have been extracted using the CNER methods that have been explained herein; they may contain variations or abbreviations of the actual material name that are used in the source article. The raw values and units present exactly how the data from the record appear in the text; normalized versions of these data are also given where they have been converted into a standard form. Stress related units are converted to Pascals and the corresponding conversion is applied to each relevant value. Metadata of each journal article are added to the corresponding data record and include the: DOI, article type, publication format, publication year, article title and journal title. The article type category varies between Elsevier and Springer publishers, but the categorization fulfils the same purpose; it includes: research article, review article, chapter, etc. This database key can be a useful filter; for example, review articles will tend to contain more data records since they often discuss more than one material or property, whereas research articles are mostly focused on a single material. Finally, the *Access Type* denotes the availability of a journal article as being either open access or not.

**Table 3:** Description of data records contained in the materials stress-strain database.

Database Key	Description	Data Type
Record Type	The property model used to extract the record. It gives the property and from where it was sourced (text or table)	String Type
Specifier	The string that determined the presence of a property mention	String Type
Compound	Extracted compound names that have been normalized. It may contain more than one entry if multiple names are given to the same compound.	List, String Type
Raw Value	Extracted value as seen in text without any normalization	String Type
Raw Units	Extracted units as seen in text without any normalization	String Type
Normalized Value	Extracted value that has been normalized using ChemDataExtractor	List, Float Type
Normalized Units	Extracted units that have been normalized	String Type
DOI	Unique identifier of the source article	String Type
Article Type	Category of article assigned by the source publisher. For example, research article or review article	String Type
Publication Format	The format by which the article is available online	String Type
Publication Year	The year that the source article became available	String Type
Article Title	The title of the source article	String Type
Journal Title	The title of the source journal	String Type
Access Type	Whether or not the source article is open-access	String Type

The content of our auto-generated materials database of stress-strain properties is provided in Table 4, in terms of its number of data records that are associated with certain mechanical properties. The full database is downloadable from the *figshare* repository^35^.

**Table 4:** Number of properties contained within the materials database of stress-strain properties.

Property	Text	Table	Total
Ultimate Tensile Strength	109,174	185,109	**294,277**
Yield Strength	90,349	125,615	**215,958**
Young’s Modulus	35,073	122,2126	**157,287**
Ductility	30,673	5510	**36,183**
Fracture Strength	5,693	10,886	**16,579**

## Technical Validation

The materials database of stress-strain properties was technically validated, to assess its usability. The evaluation metrics: precision, recall and F-score, were calculated by sampling a set of journal articles and comparing their associated auto-generated data records to manually extracted records.

The evaluation metrics of these auto-generated data records were also compared against those of the manually-extracted evaluation dataset that was previously curated for the technical validation of the materials database on yield-strength properties^23^. This comparison enabled a direct assessment of the extent by which the information-extraction methods presented in this study have improved upon those that we employed previously in this materials-domain area^23^.

### Evaluating the usability of the materials database of stress-strain properties

The usability of our auto-generated materials database of stress-strain properties was determined by assessing how well its associated information-extraction pipeline could automatically extract data from 300 open-access articles, relative to a manual data-extraction process. These 300 articles were randomly sampled from the full corpus with equal proportions stemming from Elsevier and Springer Nature publications. The entire automatic information-extraction process was emulated by a human, manually performing each step of data extraction; complete property information from each article was extracted from the 300 articles, by searching for specifiers and relevant material data from the surrounding context. The automatic information-extraction method was then used to extract data from the same 300 open-access articles. The resulting data records were compared to the manually-extracted dataset. The compound, property and units of each automatically extracted record in this evaluation database was labelled as: either a True Positive (TP), if the record exactly matched a corresponding entry in the evaluation dataset; or a False Positive (FP), if an extracted record contained incorrect information. If the automatic method did not extract a record that was in fact present in the evaluation set, a label of False Negative (FN) was assigned.

Precision is defined as the proportion of automatically extracted records that match human-extracted ones. It reflects the extent to which the information-extraction method is equivalent to a human performing the same task, whereby each automatically extracted data record is measured for its “correctness” in the sense that it corresponds to an accurate record validated by a human. Recall measures the percentage of correct records that are found in the articles by the data-extraction process; i.e., it indicates the extent to which the automatic data-extraction process is comprehensive and extracts all possible data records. These performance metrics can also be assessed using a single metric, the F-score. This is the harmonic mean of precision and recall; as such, it balances the importance of both correct data extraction (precision) and complete retrieval of information (recall). Mathematically, the evaluation metrics are given as follows:

2 $$precision=\frac{TP}{TP+FP}$$

3 $$recall=\frac{TP}{TP+FN}$$

4 $$F \mbox{-} score=2\cdot \frac{precision\cdot recall}{precision+recall}$$

These metrics were calculated independently for each facet of information being extracted: compound name, property value, and units, which are then combined to afford the precision and recall of the entire information-extraction pipeline.

The evaluation results are given in Table 5. The overall precision, recall and F-score of 82.03%, 92.13% and 86.79%, respectively. These performance statistics are comparable to those from other studies that have applied ChemDataExtractor to auto-generate materials databases containing multiple properties in other domains of materials science^16,22^.

**Table 5:** The evaluation metrics for the materials database of stress-strain properties, calculated by comparing the automatically extracted and manually extracted data.

Metric	Exact Match			Partial Match		
	Text (%)	Table (%)	Overall (%)	Text (%)	Table (%)	Overall (%)
**Precision**	73.70	88.99	82.03	78.15	95.81	87.76
**Recall**	88.05	95.19	92.13	88.66	95.52	92.61
**F-Score**	80.24	91.99	86.79	83.07	95.67	90.12

The partial-match metrics take into consideration compound names that are false positive in exact-match scoring but contain partial information that is accurate.

This technical validation process revealed that false positives for compound names would often include partially extracted compound names. For example, the complete name of a certain type of steel, *AISI 316*, may be partially extracted as *AISI 3* which still provides enough information to correctly identify the steel type. While not an exact match, partial compound names are still useful; therefore, additional evaluation metrics were calculated that accounted for them.

Table 5 shows that table extraction methods significantly outperformed text extraction methods in this study. This is a result of the highly structured nature of tables employed in the field of materials engineering through stress-strain properties. Tabulated data typically contain all relevant information with clear labels in a self-contained and data-rich format, thereby facilitating automated data extraction. Conversely, unstructured text is more nuanced with a greater diversity and complexity in how material data are conveyed; it can thus be a lot more challenging to parse accurately, as appears to be the case in this study. This disparity between text-prose and tabular data can be quantified by the 15% higher precision achieved by table-extraction methods, based on an exact-match score. The recall values for this evaluation process also reflect this trend, with 95.19% of available data being extracted from tables compared to 88.05% being extracted from text, based on an exact-match score. The overall F-score for the information-extraction process remained relatively high. This signifies that ChemDataExtractor, with the bespoke additions for stress-strain material properties presented in this work, can reproduce close to human-level performance in data extraction, at an exceedingly faster rate than any manual endeavour.

The breakdown of precision, recall, and F-score metrics for compound names, property values and their associated units are shown in Tables 6 and 7 for text and table-based information extraction, respectively. This enables a closer look at the evaluation metrics of the individual components that form a complete data record. Since the parsing rules and methods used in this work were broadly the same for each property, their metrics have been consolidated in Tables 6 and 7 for the purposes of discussion. However, a full breakdown of the evaluation metrics is given in Table 8 for the benefit of the interested reader. The total number of false negatives was used to calculate the recall for each compound name, property value and its units, since records with missing information were not extracted; therefore, the specific reason for a false negative, whether it is a missing unit, property value, or compound, cannot be accurately determined.

**Table 6:** Evaluation metrics for text extraction of compound name (extracted in full (Compound) or partially (Partial Compound)), property value (Property) and its Units.

Metric	Property	Units	Compound	Partial Compound
**Precision**	74.44	84.44	62.22	75.56
**Recall**	71.28	73.79	67.47	71.58
**F-Score**	72.83	78.76	64.74	73.51

**Table 7:** Evaluation metrics for table extraction of compound name (extracted in full (Compound) or partially (Partial Compound)), property value (Property) and its Units.

Metric	Property	Units	Compound	Partial Compound
**Precision**	94.42	100.00	72.56	93.02
**Recall**	87.50	88.11	84.32	87.34
**F-Score**	90.83	93.68	78.00	90.09

**Table 8:** All evaluation metrics recorded for the information-extraction of stress-strain properties: yield strength (YS), ultimate tensile strength (UTS), Young’s modulus (YM), fracture strength (Fracture) and ductility.

Text Extraction	Number of TP	Number of FP	Number of FN	Precision (%)	Recall (%)	F-Score (%)
Compound	112	68	54	62.22	67.47	64.74
Compound Partial	136	44	54	75.56	71.58	73.51
Property	134	46	54	74.44	71.28	72.83
YS Value	56	20	7	73.68	88.89	80.58
UTS Value	34	4	16	89.47	68.00	77.27
YM Value	24	8	28	75.00	46.15	57.14
Fracture Value	11	8	3	57.89	78.57	66.67
Ductility Value	9	6	0	60.00	100.00	75.00
Units	152	28	54	84.44	73.79	78.76
**Table Extraction**						
Compound	156	59	29	72.56	84.32	78.00
Compound Partial	200	15	29	93.02	87.34	90.09
Property	203	12	29	94.42	87.50	90.83
YS Value	62	2	6	96.88	91.18	93.94
UTS Value	32	4	2	88.89	94.12	91.43
YM Value	88	4	21	95.65	80.73	87.56
Fracture Value	17	0	0	100.00	100.00	100.00
Ductility Value	4	2	0	66.67	100.00	80.00
Units	215	0	29	100.00	88.11	93.68
**Text & Table Extraction**						
Compound	268	127	83	67.85	76.35	71.85
Compound Partial	336	59	83	85.06	80.19	82.56
Property	337	58	83	85.32	80.24	82.70
YS Value	118	22	13	84.29	90.08	87.08
UTS Value	66	8	18	89.19	78.57	83.54
YM Value	112	12	49	90.32	69.57	78.60
Fracture Value	28	8	3	77.78	90.32	83.58
Ductility Value	13	8	0	61.90	100.00	76.47
Units	367	28	83	92.91	81.56	86.86
**Overall Evaluation Metrics**						
Exact Match Text	398	142	54	73.70	88.05	80.24
Exact Match Table	574	71	29	88.99	95.19	91.99
Exact Match Overall	972	213	83	**82.03**	**92.13**	**86.79**
Partial Match Text	422	118	54	78.15	88.66	83.07
Partial Match Table	618	27	29	95.81	95.52	95.67
Partial Match Overall	1040	145	83	**87.76**	**92.61**	**90.12**

The primary source of error stems from the incorrect extraction of exact compound names, with the automatic method agreeing with human extraction from text only 62.22% of the time. While table-based extraction methods afford better precision for full compound-name identification (72.56%), CNER remains the most significant source of error. This result is a consequence of the diverse corpus employed in this study; as such, ChemdDataExtractor encounters a particularly large variety of material names in the information-extraction process. In particular, the CNER methods may therefore struggle to correctly identify longer-form compound names in this study. For example, *Polylactic acid* + *acrylonitrile butadiene styrene(60% FFF volume)*, *10% NaOH treated jute fiber reinforced epoxy composites* or *LPBF Cp-Ti* + *10.5 wt% Mo2C* are all material names that were manually identified during the technical validation stages of this work. The automatic CNER methods were able to identify components such as *Cp-Ti*, *Mo2C*, *NaOH*, *epoxy* or *Polylactic acid* but struggled to extract the long names in their entirety. This is, in part, owing to the lack of representation of these specific longer-form names in the training data used to fine-tune the SciBERT-based CNER system that was built into ChemDataExtractor version 2.1^14^. For instance, *cp-Ti* or *commercially pure Titanium* are present in the MatScholar database but not the full naming of *LPBF Cp-Ti* + *10.5 wt% Mo2C*^32,33^. The precision metrics of partially extracted compound names provide further evidence of this issue. Partial matches between automatically and manually extracted data occur in 75.56% and 93.02% of cases for text and table data, respectively. These results suggest that, while our CNER methods are able to identify materials that are included in the fine-tuning dataset of compound names, complete material names that are out-of-distribution are not fully detected, and consequently, not extracted.

Moreover, false positives in compound names arise when there are many mentions of potential chemical-entity candidates in a short text span. For example, a “materials and methods” section of a journal article is commonly used to outline the specific material composition associated with the study at hand. This will often include a base material and any additions with various processing techniques, or even comparisons to alternative materials/additives, which lead to an incorrect association of a chemical entity to the target property. Furthermore, authors may later refer to the material specifying only the additions rather than the full material name, which leads to an incorrect data extraction. Consider the example sentence, *“The UTS, YS and EI increase to 121 MPa, 105 MPa and 33% when 0.05% La is added”*; instead of the full material name being given, only the additive (*La*) is written when discussing relevant properties. To the CNER system, this is a valid entity and *La* is therefore extracted and assigned as the compound name; yet, a human can intuit that the properties stated in this sentence refer to a complete material name mentioned elsewhere in the journal article. Though ChemDataExtractor and its modifications have dedicated methods to handle compound labels, the non-standard, and often missing, labelling formats that authors often employ lead to this incorrect compound identification. Notwithstanding these issues, the CNER method was able to extract useful information with a relatively high performance.

Property extraction from table-based data demonstrates significantly higher accuracy in comparison to text. The table-based data-extraction process achieved a precision of 94.42%, showcasing near-perfect agreement with human curated data. As previously mentioned, it is more challenging to accurately parse text prose, resulting in a lower precision of 74.44% when extracting property values from textual content.

Qualitatively, the lower precision of text-based data extraction has resulted primarily from two issues. Firstly, multiple properties with the same units and similar specifiers are often mentioned in the same sentence of papers within our corpus. Shared units, similar specifiers, and similar value ranges among many properties create ambiguity for text parsers, making it challenging to distinguish between different value mentions. This issue can be illustrated using the following common example-sentence structure, *“The YS and UTS for Material X are 500 MPa and 700 MPa, respectively”*; the parser in ChemDataExtractor extracts *500 MPa* and *700 MPa* for both *UTS* and *YS* resulting in four records, two of which are incorrect. While the DepIE algorithm developed by Isazawa and Cole^15^ aimed to address this issue, the parsing method can be lenient in matching mentions of value to stress-strain property specifiers in this materials-engineering domain owing to: the relative simplicity of the property models designed herein; the relative proximity of different value mentions; and the similarity between specifiers of yield strength and ultimate tensile strength.

The second issue is that it is common for authors to mention the increase or decrease of a property value in a standalone sentence. For example, *“The Young’s modulus decreased by 150 MPa”* is common sentence structure in which all property-based components for text parsing are present: the specifier, value and unit. However, the absolute property value is not mentioned, while the extraction of *150 MPa* is considered to be a false positive.

**Fig. 4:**
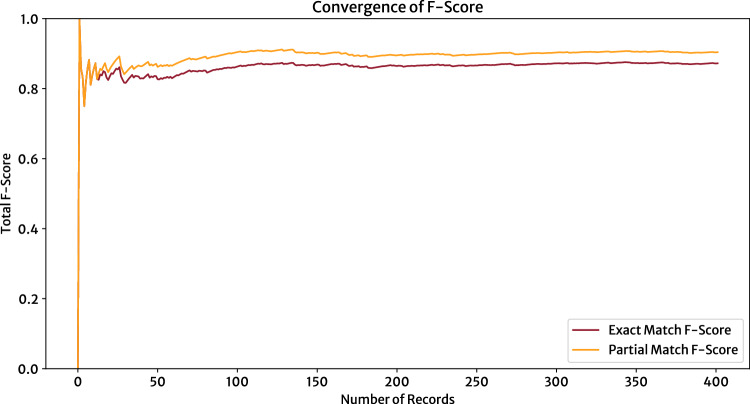
The cumulative total F-score for the materials database of stress-strain properties as a function of the number of data records being evaluated in the technical-validation process, including records that were extracted from text and tables in the source documents. The F-score calculated using only the exact-match score as well as the F-score that includes partial matches are shown. The progressive flattening of the curves suggest a convergence of F-score and that the number of records evaluated are sufficient for the purpose of ensuring a robust technical validation of our database.

To determine if our evaluation procedure had fairly assessed the performance of the information-extraction pipeline, the cumulative F-score across all records was calculated. Figure 4 shows that the F-Score for the information-extraction method employed in this technical-validation process converges as more data records are evaluated; this signifies that our evaluation dataset provides a representative and sufficient sample for the technical validation of our database. Moreover, as the number of evaluated data records increases, the F-score stabilizes, which similarly indicates a stability in the evaluation metrics.

### Evaluating the information-extraction process for stress-strain materials engineering

The performance level of the information-extraction process used in this study was further assessed, specifically in relation to that of our previous work on extracting materials and their yield-strength properties^23^. Thereby, the technical evaluation dataset manually curated by Kumar *et al*.^23^ was used to compare the veracity of the automatically generated database resulting from this study. The comparative results are detailed in Table 9.

**Table 9:** Evaluation metrics for the extraction of yield strength (YS) from text in this study compared to analogous metrics presented by Kumar *et al*.^23^.

Metric	YS (Kumar *et al*.^23^)	YS (This Study)
**Precision**	79.4%	78.1%
**Recall**	73.6%	75.9%
**F-Score**	76.4%	77.0%

Despite the added level of complexity in this study given the large number of property models and a larger corpus, a similar level of performance in information extraction was achieved. In comparison to our more focused work on yield-strength properties^23^, the precision in this study dips by 1% while its recall is higher, resulting in an overall greater F-score for this study; albeit, the overall difference is minimal. Upon closer inspection, the information-extraction methods employed in this study appear to diminish the error in extracting compound names, with a precision of 83.8% for compound name identification. Its updated CNER methodology is able to identify and extract more complicated material names, such as *Mg-8.8Sn-4.0Zn-0.9Al-0.3Na(TZA941* + *0.3Na)* which was extracted as *TZA941* in previous work^23^. Other false positives due to compound name identification have been resolved with our new CNER methods; previously extracted compounds *“‘stable HEAs”* and *“Ni3(Ti,Al)”* are now correctly extracted as *“Al5Ti5Co35Ni35Fe20”* and *“Al0.45CoCrFeNi”*, respectively.

However, our updated information-extraction methods introduced new false positives as a result of the incorrect extraction of property values. The situations in which these false positives arise are similar to what has been discussed above; namely, when multiple properties with the same units and similar specifiers are mentioned in a short span of text. By introducing more property models and modifying the parsing method, tensile strength values are sometimes incorrectly assigned to the yield strength specifier and vice versa. Nonetheless, the F-score remains high and this data-extraction process has delivered far more data on many more mechanical properties than our previous work^23^. This new materials database can therefore better inform the materials-engineering research community about stress-strain properties to facilitate the growing efforts in data-driven materials analysis in this field.

## Usage Notes

The final database has been provided in both JSON and CSV formats, allowing for easy integration into data-driven pipelines across many programming languages owing to their widely supported nature and straightforward conversion capabilities. Users can create specialized subsets of the database by filtering the provided metadata for specific journals or publication dates. Alongside this database, the exact pre-processed output from the information extraction pipeline is provided for those who wish to implement their own post-processing methodologies. The data has been made openly available and can be downloaded from *Figshare*^35^.

## Data Availability

The codebase for this study is available at https://github.com/gh-PankajKumar/chemdataextractorv2.3-stresseng. This repository includes the latest release version of ChemDataExtractor with domain-specific modifications made in this study, the webscrapers used for corpus acquisition, the extraction code and post-processing code to convert the output of extraction into suitable formats. Additional packages used in extraction have also been provided. A static version of this repository can be downloaded from *Figshare*^35^.
